# To Accept One’s Fate or Be Its Master: Culture, Control, and Workplace Choice

**DOI:** 10.3389/fpsyg.2016.00936

**Published:** 2016-06-21

**Authors:** Charis Eisen, Keiko Ishii, Yuri Miyamoto, Xiaoming Ma, Hidefumi Hitokoto

**Affiliations:** ^1^Department of Psychology, Faculty of Letters, Kobe UniversityKobe, Japan; ^2^Department of Psychology, University of Wisconsin–MadisonMadison, WI, USA; ^3^Kokoro Research Center, Kyoto UniversityKyoto, Japan

**Keywords:** culture, agency, choice, workplace preferences, desire for control

## Abstract

Utilizing three student (Study 1) and non-student samples (Study 2), we examined cultural differences in workplace choice for North Americans, Germans, and Japanese. We focused on the desire for control as a potential mediator (i.e., the underlying mechanism) to explain cultural differences in this important life decision. Given culturally divergent embodiments of independent vs. interdependent models of agency, we expected and found that, compared to North Americans and Germans, Japanese were more likely to prefer a workplace with a payment system that maintains social order rather than one that rewards individual achievement. Furthermore, we found that Japanese tend to give greater consideration to family opinions in their choice of workplace. As predicted, desire for control (i.e., the motivation to have control over various events) was stronger for North Americans and Germans than Japanese, and explained cultural differences in choice of workplace.

## Introduction

Previous studies have documented cultural differences in decision-making and the apparent motivations for these decisions (Heine and Lehman, 1997; Iyengar and Lepper, 1999; Kim and Markus, 1999; Kim and Drolet, 2003; Kitayama et al., 2004; Savani et al., 2008). Whereas Westerners tend to venerate the principle of liberal, unhindered choice, East Asians place greater importance on choice made in the context of maintaining harmonious social relationships. Extending the previous findings, the present research addresses whether these cultural differences can be observed in choices that have significant implications in individuals’ real lives, such as the choice of one’s workplace (hereafter, referred to as *workplace choice*). Moreover, compared to the large amount of evidence on cultural differences in choice, relatively little is known about mechanisms underlying these divergent preferences. In the present research, we explore a possibility that cultural differences in choice are associated with different levels of desire for control over events. Focusing on workplace choice, we specifically examined whether the desire for control, which is expected to be higher in Western people such as North Americans and Germans than in East Asians such as Japanese, would lead people to choose a workplace that emphasizes individual achievements, and conform more to their own career aspirations than to the opinions of family members.

### Culture and Choice

While the basic need for choice may be biologically motivated and hence universal, the definition of choice and related behavioral tendencies can be altered as a result of cultural norms (Leotti et al., 2010; Stephens et al., 2011). Indeed, European Americans are more likely than East Asians to view choice as an expression of internal attributes (Kim and Sherman, 2007). Moreover, individual choice seems to have a greater importance for Westerners than for Easterners. For example, Savani et al. (2008) found that, compared to North Americans, Indians were slower to make choices and less motivated to express individual preferences in their choices. Similarly, Iyengar and Lepper (1999) experimentally demonstrated that European American children were more motivated when they chose by themselves, whereas Asian American children were more motivated when their mother chose on their behalf. When people hold a predominantly independent self-concept, like in many Western cultures, listening to the opinions of close others is often understood as an obligation that snatches the freedom out of choice, or as an unpleasant obligation, whereas for people holding predominantly interdependent self-concepts, like in many East Asian cultures, well-intentioned and supportive advice from significant others can have a motivating function (Fu and Markus, 2014).

### Culture and Workplace Choice

The purpose of the present research is to extend the body of research on culture and choice by focusing on workplace choice and to explore a possible underlying psychological mechanism for any observed cultural differences. Evidence on cultural differences in factors people are likely to consider in their workplace decision is still limited (but see Lee and Ramaswami, 2013). Brew et al. (2001), for example, collected data from Australian students of European and Chinese descent, and investigated the students’ workplace choice. The researchers’ career choice vignette involved choosing to follow either one’s own career aspirations or family expectations. Chinese students were more inclined to respect family wishes, whereas European students were more inclined to take responsibility and make their own decisions about their careers. Likewise, researchers have argued that parental and family influences on one’s job decisions should be stronger in East Asian countries (e.g., Phillips et al., 2001). In the present research, we tested North American, German, and Japanese students and examined whether the findings of Brew et al. (2001) can be extended to these three cultures.

We also focused on another important type of workplace decision: the choice over a performance-based vs. a seniority-based payment system. Because the primary goal of companies is to increase profit, they are likely to allocate rewards to employees who make great efforts and excel at work. These performance-based rewards signal to employees that their work is recognized and valued, and thus functions as a work motivation. Alternatively, companies can reward their employees according to seniority, i.e., the time they have spent working for this company. Doing so binds employees to the organization as staying loyal will pay off in the long run; a seniority-based system encourages a stable social order, and enables a strong identification with the organization and colleagues (Rusbult et al., 1995).

Previous research has found cross-cultural differences in reward allocation behavior (for an overview, see Fischer and Smith, 2003) and the perceived fairness of seniority-based and performance-based payment systems (Fischer and Smith, 2004). Furthermore, comparing these different types of payment systems, Brown and Reich (1997) argued for a tight link between the organizational system and the context within which it is located. Congruently, the literature on the fit between people and work environments [P–E fit] suggests that P–E fit depends on cultural context (Chatman, 1989; Kristof, 1996; see also Lee and Ramaswami, 2013 for a recent review). In the recruitment process, for example, North American firms put more emphasis on the match between an individual’s skills and the job’s requirements, whereas Japanese firms emphasize similarities between individuals and organizations (Sekiguchi, 2006). Indeed, employees in individualistic cultures tend to receive rewards based on their individual contribution to the company’s goals, while employees in collectivistic cultures such as those of East-Asia, tend to receive rewards based on seniority and adhering to group-oriented collectivistic values (Erez, 2000). Accordingly, analyzing career orientations in Canadian and Japanese students, Firkola and Tiessen (1998) described cultural differences in the general expectation about management: Japanese students tended to expect responsibilities to come over time (i.e., seniority), and not, as Canadian students, as a consequence of ambition or performance. Based on this research background, we examined whether performance-based or seniority-based payment systems are preferred in people’s workplace choice across three cultures.

In the present research, we prepared two scenarios, one of which was created by adapting the career vignette by Brew et al. (2001) that pitted one’s own career aspirations vs. family expectations. We also developed a vignette that pitted performance-based vs. seniority-based payment systems. Given previous findings, we expected that Japanese would be more likely than North Americans and Germans to attach importance to family expectations in their workplace choice. Further, we expected North Americans and Germans to be more likely than Japanese to prefer a performance-based payment system.

### The Desire for Control as a Potential Mediator of Cultural Differences in Workplace Choice

In addition to elucidating cultural differences in workplace choice, the second purpose of the present research was to explore a psychological mechanism underlying cultural differences. Although various studies have shown cultural differences in choice, relatively little is known about the potential mechanisms responsible for these differences. Here, we focus on the desire for control as a mediator that may, at least partly, explain cultural differences in workplace choice.

In Western cultures, where independence and autonomy of the self are stressed, personal control is often seen as a virtue (Weisz et al., 1984). Consistent with this perspective, North Americans are more likely than Japanese to report that they have influenced the people and events in their lives (Morling et al., 2002).

We expect cultural differences in the desire for control to influence workplace choice because aspects of workplaces present different opportunities to directly exercise control. Nevertheless, no studies, to our knowledge, have examined the association between the desire for control and workplace choice cross-culturally. For instance, while a performance-based payment system allows workers to directly influence how much money they have in their pocket, a seniority-based payment system offers no room to control one’s salary personally. Moreover, when it comes to close others’ influence on one’s decisions, it is supposable that people who desire to control the environment themselves are more likely to follow personal beliefs and goals rather than following family members’ opinions.

We thus expected that culturally different degrees of desirability for control would explain the effects of culture on workplace choice. Specifically, we measured individual differences in the general level of motivation for control with the desirability of control scale (Burger and Cooper, 1979).

### Present Research

The present research examines cultural differences in workplace choice and a possible underlying mechanism responsible for these differences - desire for control. We hypothesize that Japanese prefer a workplace with a seniority-based payment system that emphasizes belonging, whereas Westerners prefer a workplace with a performance-based payment system that rewards individual achievement (Hypothesis 1). We also hypothesize that Japanese place greater emphasis on family member opinions regarding workplace choice than Westerners (Hypothesis 2). Moreover, we expect that individual differences in the desire for control mediate the effect of culture on both payment system preference and the importance of family influence regarding the choice of one’s workplace (Hypothesis 3).

In the present research, adopting the method of triangulation (Medin et al., 2007), we employed three cultural group comparisons and chose North Americans and Germans as our Westerners groups. Past studies have shown that North Americans are more independent than Germans in various implicit measurements of interdependence, including context sensitivity and predictors of happiness, while Japanese are less independent than both Western groups (Kitayama et al., 2009). These results suggest that not only Western cultural heritage but also the history of voluntary settlement featured in North American cultures foster the ethos of independence as proposed by the voluntary settlement hypothesis (Kitayama et al., 2006, 2009). Given this, in addition to the three hypotheses regarding East–West differences, we expect to find systematic differences between North Americans and Germans in terms of workplace decisions: as a result of stronger independence, compared to Germans, North Americans’ preference for a performance-based system might be stronger, and family opinions might be considered less.

## Study 1

### Materials and Methods

#### Ethics Statement

The study was reviewed and approved by the Experimental Research Ethics Committee at the Graduate School of Humanities, Kobe University. Participants provided a written informed consent at the beginning of the study. All responses were confidential.

#### Materials

All materials were initially developed in English. They were then translated into German and Japanese, and back-translated into English by independent translators. Finally, back-translated materials were checked for consistency with the original English language materials. This process allowed us to ensure for the uniformity of materials across cultures.

##### Workplace Choice Vignettes

We constructed two vignettes following the procedure of previous research investigating individualist-collectivist differences in decision-making (Brew et al., 2001). To the extent possible, we used concrete as opposed to abstract scenarios; we did this to avoid potential confounds inherent to cross-cultural comparisons (Peng et al., 1997). The vignettes (see Appendices A and B) described (i) a workplace choice concerning a payment system, and (ii) a workplace choice concerning family influence. In particular, the first vignette described a workplace with a seniority-based payment system, which connected salary to the employee’s hierarchical standing in the company (i.e., how many years he or she had worked for the company), and a workplace with a performance-based payment system, which connected salary to individual achievement. The second vignette described a workplace consistent with the participant’s career aspirations but inconsistent with familial expectation, and a workplace where the reverse was true. After each vignette, adopting a 9-point bi-polar scale used in Brew et al. (2001), we measured preference for workplace (1: the workplace with the interdependent emphasis; 9: the workplace with independent emphasis). Furthermore, after each vignette, six questions assayed for the reasoning behind a particular workplace preference, again measured on a 9-point scale (1: strongly disagree, 9: strongly agree). There were three questions regarding interdependent reasons (e.g., “The seniority-based payment system gives me a feeling of security,” “My family knows what is best for me”) and three questions regarding independent reasons (e.g., “The performance-based payment system motivates me,” “It is important for my future happiness that I make my own decisions about my career”).

#### The Desirability of Control Scale

This scale was developed by Burger and Cooper (1979) to measure the level of “motivation to control the events in one’s life” (p. 381). The scale consists of 20 items (e.g., “I enjoy having control over my own destiny”). Participants were asked to indicate the extent to which they agreed with each item on a 7-point Likert-type scale (1: strongly disagree, 7: strongly agree). Reasonable reliabilities were confirmed in each of the three cultures (North Americans: α = 0.81, Germans: α = 0.73, Japanese: α = 0.80). Confirmatory factor analyses revealed the original five-factor structure fits the data moderately well, CFI = 0.75, RMSEA = 0.09 (North Americans: CFI = 0.72, RMSEA = 0.10, Germans: CFI = 0.66, RMSEA = 0.10, Japanese: CFI = 0.81, RMSEA = 0.08).

### Participants and Procedure

One hundred and thirty-one European American students at the University of Wisconsin-Madison (37 males and 94 females), 86 German students at the University of Mannheim (31 males and 55 females), and 81 Japanese students at Kobe University (44 males and 37 females) participated in the study. North American students were given course credit for their participation. German participants volunteered without reward. Japanese students were paid 500 yen (about $5).

Participants first read one of the two vignettes, indicated their preference for one of two workplaces, and answered six questions regarding the reasons for their choice. Participants next read the remaining vignette, and answered the associated questions. Finally, participants completed the measure of desire for control. The study took approximately 20 min to complete.

### Dependent Variables

We created an index representing participants’ emphasis on independent values or interdependent values regarding workplace choice by calculating the mean rating of their choice and their agreement to the items measuring independent and interdependent motivations for their choice. This index served as our primary dependent variable. Higher ratings on the index indicate more agreement with independent values (i.e., preference for a performance-based system and indifference to family approval), while lower ratings indicate more agreement with interdependent values (i.e., preference for a seniority-based payment system or consideration of family opinions). Reliability analyses confirmed the validity of this procedure (payment system: α_German_ = 0.77, α_Japanese_ = 0.81, α_NorthAmerican_ = 0.76; family influence: α_German_ = 0.72, α_Japanese_ = 0.64, α_NorthAmerican_ = 0.68).

### Results and Discussion

There were significant age differences among the three cultures, *F*(2, 294) = 44.58, *p* < 0.001, *η*^2^ = 0.23. Also, regression analyses showed that the preference for a performance-based payment system and the influence of family decrease with age, *b* = -0.09, *SE* = 0.05, *t*(293) = -1.93, *p* = 0.05, and *b* = 0.08, *SE* = 0.04, *t*(295) = 2.11, *p* = 0.04, respectively. We thus controlled for age in the following analysis. **Table 1** presents descriptive statistics for each cultural group.

**Table 1:** Descriptive statistics for the variables for each cultural group in Study 1.

	Japanese		Germans		North Americans	
	Mean	*SD*	Mean	*SD*	Mean	*SD*
Age	19.59	0.89	21.15	2.38	18.93	1.54
Payment system	5.35	1.63	6.04	1.61	6.78	1.28
Family influence	6.18	1.33	7.02	1.20	6.54	1.17
Desire for control	4.39	0.71	4.97	0.55	4.95	0.65

*In the payment system vignette, 1 represents the seniority-based system and 9 represents the performance-based system; In the family influence vignette, 1 represents the workplace consistent with family expectations and 9 represents the workplace inconsistent with family expectations. Concerning the interpretation of the desire for control values, the higher the value the higher the desire for personal control.*

#### Payment System

An analysis of covariance (ANCOVA) showed a main effect for culture, *F*(2, 291) = 21.99, *p* < 0.001, *η*^2^ = 0.13. Consistent with Hypothesis 1, compared to Japanese students, both Germans and North Americans preferred the performance-based payment system, *t*(291) = 3.05, Tukey’s adjusted *p* = 0.007, *r* = 0.18, and *t*(291) = 6.63, Tukey’s adjusted *p* < 0.001, *r* = 0.36, respectively. Moreover, North Americans preferred the performance-based payment system more strongly than Germans did, *t*(291) = 2.80, Tukey’s adjusted *p* = 0.01, *r* = 0.16.

#### Family Influence

An ANCOVA showed a significant main effect of culture, *F*(2, 293) = 7.89, *p* < 0.001, *η*^2^ = 0.05. Consistent with Hypothesis 2, compared to Germans, Japanese were more strongly influenced by their parents’ opinions about companies, *t*(293) = 3.95, Tukey’s adjusted *p* < 0.001, *r* = 0.22. The same tendency was found in the comparison between Japanese and North Americans, although the difference was marginally significant, *t*(293) = 2.28, Tukey’s adjusted *p* = 0.06, *r* = 0.13. Moreover, there was no significant difference between German and North Americans in the level of family influence, *t*(293) = 2.02, Tukey’s adjusted *p* = 0.11, *r* = 0.12.^1^

#### Underlying Mechanism: The Desire for Control

As displayed in **Table 1**, Germans and North Americans scored higher on the desirability of control scale than Japanese, *F*(2, 295) = 22.99, *p* < 0.001, *η*^2^ = 0.13, *t*(295) = 5.81, *p* < 0.001, *r* = 0.32 for the German – Japanese contrast, *t*(295) = 6.17, *p* < 0.001, *r* = 0.34, for the North American – Japanese contrast. No significant difference was found between Germans and North Americans, *t*(295) = 0.20, *p* = 0.85. These results are in line with prior research that found North Americans want to influence their surroundings stronger than Japanese (e.g., Morling et al., 2002).

**FIGURE 1:**
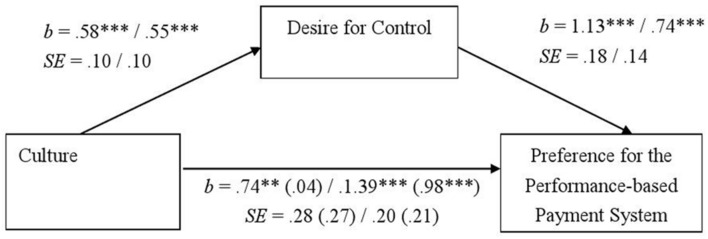
**The desire for control as a mediator of the cultural differences in students’ payment system preferences in Study 1.** Unstandardized coefficients and standard errors are shown. Coefficients and standard errors regarding the relationship between culture and the preference for the performance-based payment system after controlling the desire for control are given in parentheses. Left: Japanese (0)–German (1) comparison. Right: Japanese (0)–North American (1) comparison. *^∗∗^p <* 0.01, *^∗∗∗^p <* 0.001.

To see the mechanism that underlies the cultural differences, we investigated whether the desire for control mediates the differences between Japanese and Westerners (Germans and North Americans, respectively) in their workplace choice. We dummy coded culture (Japanese = 0 vs. German = 1, and Japanese = 0 vs. North Americans = 1) and conducted a linear regression analysis with culture as the predictor and payment system as the dependent measure. Compared to Japanese, Germans and North Americans preferred a performance-based system, *b* = 0.74, *SE* = 0.28, *t*(162) = 2.69, *p* = 0.008, and *b* = 1.39, *SE* = 0.20, *t*(208) = 6.67, *p* < 0.001, respectively, and were higher in the desire for control, *b* = 0.58, *SE* = 0.10, *t*(162) = 5.86, *p* < 0.001, and *b* = 0.55, *SE* = 0.10, *t*(208) = 5.60, *p* < 0.001, respectively. When both culture and the desire for control were entered simultaneously as predictors in a regression analysis, the desire for control significantly predicted the preference for performance-based system, *b* = 1.13, *SE* = 0.18, *t*(161) = 6.28, *p* < 0.001 for the German contrast, *b* = 0.74, *SE* = 0.14, *t*(207) = 5.42, *p* < 0.001, for the American contrast, while the effect of culture became non-significant in the German contrast, *b* = 0.04, *SE* = 0.27, *t*(161) = 0.14, *p* = 0.89. On the other hand, the effect of culture was still significant after controlling the desire for control in the American contrast, *b* = 0.98, *SE* = 0.21, *t*(207) = 4.67, *p* < 0.001. A bootstrap analysis with 95% (bootstrap sample = 10000), which was conducted following Preacher and Hayes (2004), revealed a significant indirect effect (confidence intervals = [0.40, 0.94] and [0.22, 0.65] for German and American contrasts, respectively). Thus, as shown in **Figure 1**, the desire for control mediated the effect of culture on payment system preferences in the Japanese–German comparison, and we found a partial mediation in the Japanese–North American comparison.

**FIGURE 2:**
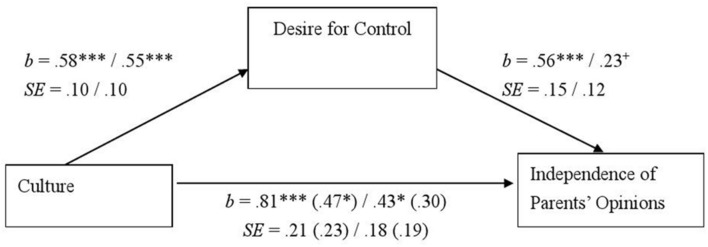
**The desire for control as a mediator of the cultural differences in the degree of students’ consideration of their parents’ opinions in Study 1.** Unstandardized coefficients and standard errors are shown. Coefficients and standard errors regarding the relationship between culture and independence of parents’ opinions after controlling the desire for control are given in parentheses. Left: Japanese (0)–German (1) comparison. Right: Japanese (0)–North American (1) comparison. ^+^*p <* 0.10*, ^∗^p <* 0.05, ^∗∗∗^*p* < 0.001.

Next, we conducted a mediation analysis using the above described procedure concerning family influence. Compared to Japanese, Germans and North Americans were less influenced by their parents’ opinions, *b* = 0.81, *SE* = 0.21, *t*(164) = 3.78, *p* < 0.001, and *b* = 0.43, *SE* = 0.18, *t*(208) = 2.40, *p* = 0.02, respectively. The desire for control significantly predicted family influence, *b* = 0.56, *SE* = 0.15, *t*(163) = 3.70, *p* < 0.001, while the effect of culture was still significant in the German contrast, *b* = 0.47, *SE* = 0.23, *t*(163) = 2.06, *p* = 0.04. On the other hand, the desire for control somewhat predicted the influence of family, *b* = 0.23, *SE* = 0.12, *t*(207) = 1.82, *p* = 0.07, and the effect of culture became non-significant, *b* = 0.30, *SE* = 0.19, *t*(207) = 1.59, *p* = 0.11 in the American contrast. A bootstrap analysis with 95% (bootstrap sample = 10000) revealed a significant indirect effect in the German contrast (confidence intervals = [0.12, 0.56]), although the American contrast was non-significant (confidence intervals = [-0.03, 0.30]) (**Figure 2**). This suggests that the desire for control partially mediates cultural differences in family influence on workplace choice, although the mediation effect is weak in the Japanese–American comparison. Taken together, the results partially support our third hypothesis.

## Study 2

Study 2 investigated the same three hypotheses concerning individual differences in workplace choice and their underlying mechanism in a sample consisting of working adults. While students usually do not have work experience and might therefore choose their workplace in accordance with their conception of an ideal workplace based on information they get from other people or the media, non-student adults should have a more realistic conception since they have actual work experience. Further, previous research suggests that people with higher socio-economic status (SES) exert more choice than those with lower SES (e.g., Snibbe and Markus, 2005; Stephens et al., 2011). To separate the effects of SES from those of culture, we included measurements of and controlled for objective and subjective SES in the following analyses. Moreover, how participants were rewarded for their participations differed across cultures in Study 1, which might influence cultural differences in workplace choice and the desire for control. To solve these issues and confirm the validity of the findings of Study 1, we used online survey services, administered the same questionnaires to non-student adults in the U.S., Germany, and Japan, and examined whether cultural differences in workplace choice extend to them as well.

### Materials and Methods

#### Ethics Statement

The study was reviewed and approved by the Experimental Research Ethics Committee at the Graduate School of Humanities, Kobe University. Participants were initially presented with an informed consent form. By agreeing to the conditions stated in this form and indicating their consent to participate in the study, the instructions of the study started. All responses were confidential.

#### Materials

The materials were the same as study one with two exceptions. First, we changed the family influence vignette so that it opposes workplaces with which the family (and not the parents) agrees or disagrees. Second, we included questions on objective and subjective SES. We assessed objective SES by asking participants about their education (i.e., “What is your highest educational attainment?”) and their yearly income (i.e., “What is your income?”). Educational attainment was assessed on a 6-point scale (1 = some high school, 2 = completed high school, 3 = some college, 4 = completed college, 5 = some post graduate, and 6 = post graduate degree). The level of income was assessed on a 8-point scale (1 = less than $20,000, 2 = $20,000–$40,000, 3 = $40,000–$60,000, 4 = $60,000–$80,000, 5 = $80,000–$100,000, 6 = $100,000–$120,000, 7 = $120,000–$140,000, and 8 = more than $140,000). The questionnaires asked participants to indicate their income in the currency of their country, respectively. Thus, North American participants chose between the options explained above in USD, German participants indicated their income in the equivalent amount of Euro, and Japanese participants reported their income in JPY. The two measures were standardized and averaged for each of the cultures to produce an indicator of objective SES. To measure subjective SES, participants were presented with a picture of a 10-rung ladder and asked to place themselves on the ladder based on where they stand compared to other people in their country (adopted from Adler et al., 1994). They were assigned scores ranging from 1 (lowest rung) to 10 (highest rung).

### Participants and Procedure

Three hundred and forty-three non-student adults from three different cultures participated in this study: 123 North Americans (54 men, 69 women), 114 Germans (63 men, 51 women), and 106 Japanese (53 men, 53 women). They came from different cities all over the three countries and worked in various fields. Participants were recruited using online survey services, specifically Amazon’s Mechanical Turk for Americans, WorkHub for Germans, and Micromill for Japanese. All three survey companies have a pool of internet users at their disposal and rewarded the participants with a small monetary compensation adapted to the national norms, respectively. The procedure was the same as Study 1, however, participants completed these additional SES questions, and the study was conducted online. The study took approximately 20 min to complete.

### Dependent Variables

As in Study 1, the dependent variable was the mean value of the decision tendency for a payment system and familial influence, and the agreement to independent and interdependent reasons, respectively (payment system: α_German_ = 0.76, α_Japanese_ = 0.74, α_NorthAmerican_ = 0.72; family influence: α_German_ = 0.80, α_Japanese_ = 0.74, α_NorthAmerican_ = 0.67).

### Results and Discussion

**Table 2** shows the descriptive statistics for the variables for each cultural group. There were significant differences among the three cultures in educational attainment, *F*(2, 340) = 4.66, *p* = 0.01, *η*^2^ = 0.03, yearly income, *F*(2, 340) = 3.74, *p* = 0.02, *η*^2^ = 0.02, and subjective SES, *F*(2, 340) = 12.02, *p* < 0.001, *η*^2^ = 0.07, whereas mean age did not differ significantly across cultures, *F*(2, 340) = 1.99, *p* = 0.14, *η*^2^ = 0.01. We controlled for age, objective SES (i.e., the index created by standardizing educational attainment and income), and subjective SES in the following analysis.

**Table 2:** Descriptive statistics for the variables for each cultural group in Study 2.

	Japanese		Germans		North Americans	
	Mean	*SD*	Mean	*SD*	Mean	*SD*
Age	34.83	8.44	34.20	6.34	36.74	13.89
Educational attainment	3.23	1.18	3.64	1.02	3.62	1.18
Yearly income	1.96	1.10	2.41	1.33	2.39	1.49
Subjective SES	4.36	1.83	5.53	1.76	4.79	1.80
Payment system	4.56	1.45	4.90	1.69	5.82	1.68
Family influence	5.04	1.27	6.14	1.41	5.88	1.54
Desire for control	4.16	0.58	4.96	0.61	4.95	0.74

*In the payment system vignette, 1 represents the seniority-based system and 9 represents the performance-based system; In the family influence vignette, 1 represents the workplace consistent with family expectations and 9 represents the workplace inconsistent with family expectations. Concerning the interpretation of the desire for control values, the higher the value the higher the desire for personal control.*

#### Payment System

An ANCOVA showed that the main effect of culture was significant, *F*(2, 337) = 19.94*, p* < 0.001, *η*^2^ = 0.11. Consistent with Hypothesis 1, North Americans preferred the performance-based payment system stronger than Japanese, *t*(337) = 5.85, Tukey’s adjusted *p* < 0.001, *r* = 0.30. Germans scored in between those groups, demanding the performance-based payment system significantly less than North Americans, *t*(337) = 4.73, Tukey’s adjusted *p* < 0.001, *r* = 0.25, whereas they preferred the performance-based payment system stronger than Japanese, although the difference was not significant, *t*(337) = 1.09, Tukey’s adjusted *p* = 0.52, *r* = 0.06. Given the result of Study 1 indicating that German students preferred the performance-based payment system stronger than Japanese students, this unexpected finding might reflect uncontrolled differences of working experiences and careers between the Japanese and German adult samples.

#### Family Influence

An ANCOVA showed a main effect of culture, *F*(2, 337) = 18.70, *p* < 0.001, *η*^2^ = 0.10. Consistent with Hypothesis 2, compared to Germans and North Americans, Japanese were significantly stronger influenced by their family’s opinions, *t*(337) = 5.95, Tukey’s adjusted *p* < 0.001, *r* = 0.31, and *t*(337) = 4.36, Tukey’s adjusted *p* < 0.001, *r* = 0.23, respectively. There was no significant difference in the level of family influence between Germans and North Americans, *t*(337) = 1.90, Tukey’s adjusted *p* = 0.14, *r* = 0.10.^2^

#### Underlying Mechanism: The Desire for Control

Reasonable reliabilities were confirmed for the desirability of control scale in the three cultures (North Americans: α = 0.81, Germans: α = 0.75, Japanese: α = 0.78). Confirmatory factor analyses revealed the original five-factor structure fits the data moderately well, CFI = 0.79, RMSEA = 0.10 (North Americans: CFI = 0.75, RMSEA = 0.11, Germans: CFI = 0.77, RMSEA = 0.10, Japanese: CFI = 0.70, RMSEA = 0.11). As in Study 1, Germans and North Americans scored higher than Japanese on the scale, *F*(2, 340) = 55.14, *p* < 0.001, *η*^2^ = 0.24, *t*(340) = 9.14, *p* < 0.001, *r* = 0.44, for the German–Japanese contrast, *t*(340) = 7.22, *p* < 0.001, *r* = 0.36, for the North American–Japanese contrast. There was no significant difference between Germans and North Americans, *t*(340) = 0.09, *p* = 0.93, *r* = 0.01.

**FIGURE 3:**
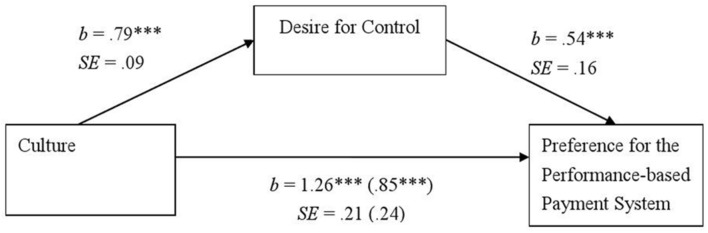
**The desire for control as a mediator of the cultural differences in adults’ payment system preferences in Study 2.** Unstandardized coefficients and standard errors are shown. A coefficient and a standard error regarding indicating the relationship between culture and the preference for the performance-based payment system after controlling the desire for control are given in parentheses. Japanese (0)–North American (1) comparison. *^∗∗∗^p <* 0.001.

A mediation analysis comparing Japanese (0) and Germans (1) was only performed regarding family influence because we did not find a significant difference between the two cultures in payment system preferences. As for the Japanese (0)–North American (1) comparison, we tested the effect of the desire for control as a mediator on both payment system preferences and family influence. As mentioned, compared to Japanese, German and North Americans scored higher on the desire for control scale, *b* = 0.80, *SE* = 0.08, *t*(215) = 9.97, *p* < .001, and *b* = 0.79, *SE* = 0.09, *t*(224) = 8.96, *p* < 0.001, respectively. Moreover, desire for control significantly predicted payment system preferences in the comparison between Japanese and North Americans, *b* = 0.54, *SE* = 0.16, *t*(223) = 3.47, *p* < 0.001. North Americans were more inclined toward a performance-based payment system than were Japanese, *b* = 1.26, *SE* = 0.21, *t*(224) = 5.99, *p* < 0.001, however, this effect became weaker after controlling for desire for control, *b* = 0.85, *SE* = 0.24, *t*(223) = 3.47, *p* < 0.001 (**Figure 3**). A bootstrap analysis with 95% (bootstrap sample = 10000) revealed a significant indirect effect (confidence intervals = [0.15, 0.77]).

**FIGURE 4:**
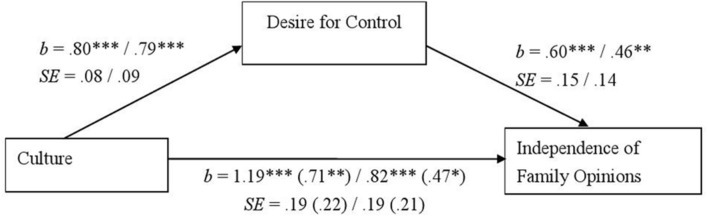
**The desire for control as a mediator of the cultural differences in adults’ degree of consideration of family members’ opinions in Study 2.** Unstandardized coefficients and standard errors are shown. Coefficients and standard errors regarding the relationship between culture and independence of parents’ opinions after controlling the desire for control are given in parentheses. Left: Japanese (0)–German (1) comparison. Right: Japanese (0)–North American (1) comparison. *^∗^p <* 0.05, *^∗∗^p <* 0.01, and *^∗∗∗^p <* 0.001.

Furthermore, desire for control significantly predicted family influence in the German – Japanese contrast, *b* = 0.60, *SE* = 0.15, *t*(214) = 3.90, *p* < 0.001, and in the American – Japanese contrast, *b* = 0.46, *SE* = 0.14, *t*(223) = 3.24, *p* = 0.001. Compared to Japanese, Germans and North Americans were less influenced by their families’ opinions, *b* = 1.19, *SE* = 0.19, *t*(215) = 6.16, *p* < 0.001, and *b* = 0.82, *SE* = 0.19, *t*(224) = 4.32, *p* < 0.001, respectively. This effect became weaker after controlling for desire for control, *b* = 0.71, *SE* = 0.22, *t*(214) = 3.19, *p* = 0.002, and *b* = 0.47, *SE* = 0.21, *t*(223) = 2.20, *p* = 0.03, for German and American contrasts, respectively (**Figure 4**). A bootstrap analysis with 95% (bootstrap sample = 10000) revealed a significant indirect effect (confidence intervals = [0.23, 0.73] and [0.13, 0.61] for German and American contrasts, respectively). In sum, except for the Japanese–German comparison regarding payment system preferences, cultural differences in payment system preferences and family influence appeared and were partially mediated by the desire for control. In spite of the non-significant indirect effect on family influence in the comparison of Japanese and American students in Study 1, the indirect effect turned to be significant in Study 2 testing non-student adults. The results of the mediation analyses support Hypothesis 3.

## General Discussion

In two studies with students (Study 1) and non-student adults (Study 2), we found cultural differences in preferred workplace characteristics and close others’ influence on workplace choice. In line with our first hypothesis, North Americans and Germans were more likely than Japanese to choose a workplace with a performance-based system over a seniority-based system. Moreover, consistent with our second hypothesis, we found Japanese place more weight on family members’ opinions regarding workplace choice than Westerners. Perhaps most importantly, consistent with our third hypothesis, we found evidence that individual differences in the desire for control mediate these cultural differences.

Our results indicate that people’s individual preferences for work-related payment-systems depend on their cultural background. Japanese value a system, which binds employees to the organization, and thus encourages them to identify strongly with their workplace. By contrast, Westerners prefer a system that rewards them according to their individual contribution to the company’s success. Moreover, North Americans were more likely than Germans to prefer a system that focuses on individual achievement. This is consistent with previous findings proposing greater emphasis on individual achievement and self-promotion in North America, compared to Germany (Kitayama et al., 2009).

Previous studies have shown that family member’s influence, which may be seen as a restriction of personal freedom in the West, may be internalized and act as powerful motivational force for people in East Asia (Iyengar and Lepper, 1999; Brew et al., 2001; Savani et al., 2010; Fu and Markus, 2014). Our investigation expands on these findings, as it suggests that, even for very important personal decisions such as workplace choice, the value attached to others’ opinions differs across cultures, and is greater for Japanese than for Westerners. Unexpectedly, we found that Germans were less likely than North Americans to consider family opinions in their workplace choice. This suggests a stronger tendency to condemn social compliance for Germans than for North Americans. However, as we did not measure any value differences between the Western cultures, we refrain from conjecture as to why this difference emerged.

Regarding the underlying mechanism for the abovementioned cultural differences, the present study, is to our knowledge, the first to show that the desire for control, which is higher in North Americans and Germans than in Japanese, mediates cultural differences in workplace choice. Our results suggest that Japanese do not wish to exercise influence as strongly as Westerners because their cultural norms encourage the individual to adjust to his/her social surroundings instead of controlling them. Such a cultural difference in the striving for personal control partly explains the extent to which people prefer a workplace with a performance-based payment system and consider family recommendations. Our exploration of an underlying mechanism to explain cultural variation, contributes to the cross-cultural research on agency, and choice by showing that culture (i.e., Japanese vs. Westerners) and workplace choice (i.e., a workplace consistent with interdependent values vs. independent values) is not related in a simple binary fashion. Instead, our research suggests that culturally distinct workplace preferences are, at least partially, a consequence of individual differences in desire for control.

The present study has some noteworthy limitations. Firstly, it was completely based on questionnaires. While we constructed scenarios as realistically as possible (in accordance with the recommendation of Peng et al., 1997), and while it is likely that participants’ actual decisions would be very similar to their self-reported answers, future research should employ methods with more concrete behavioral outcomes. Secondly, this research assumes that a seniority-based payment system reflects hierarchical social order and highlights group membership. While this may be true, seniority-based pay might also be seen as a second order merit based reward: more senior members are more likely to have greater wisdom and experience, to be more attached and committed to the organization and thus, to enhance productivity and workflow (Rusbult et al., 1995). Therefore, it might be the indirect merit based reward function in addition to the representation of the social order and belonging to an organization that made the seniority-based system more attractive for Japanese than for Westerners. Concerning the generalizability of findings, it is important to keep in mind that the three cultures studied are examples of democratic, developed, and capitalistic societies. Thus, some of the assumptions implied in this research may not apply to developing or less democratic societies. For example, the magnitude of the pay might play a bigger role than the nature of the reward system in developing countries, and the comparison of payment systems might thus be only a secondary criterion. Finally, as the research shows some (minor) differences between the two Western societies, it is important to be aware of differences that might exist between cultures that are commonly perceived to be similar, and to be mindful when generalizing across national cultures.

To achieve a fuller understanding of workplace preferences and the mechanisms shaping cultural differences in these preferences, we believe it is important for researchers to continue this line of investigation. Although we found that Westerners are more likely than Japanese to base their choice of workplace solely on individual preference due to relatively higher desire for control, other aspects of culture could also contribute to the differences in workplace choice. One of these other aspects, which we consider worth investigating, is uncertainty avoidance. In societies with high uncertainty avoidance, which is an index of the degree to which the individuals of a society feel uncomfortable with uncertainty and ambiguity (Hofstede et al., 2010), the seniority-based system was found to be more widespread than the performance-based system of payment (Schuler and Rogovsky, 1998). In line with this study’s findings, Japans’ scores on this dimension are higher than the United States’, and the scores of Germany are in the middle of the two cultures (Hofstede et al., 2010). Therefore, the relationship between uncertainty avoidance and workplace choice remains to be tested in future research.

Moreover, to get a broader understanding of culture and workplace decisions, further research should examine the degree to which observed workplace preferences represent widespread cultural norms. Many cultural differences are explained by unpacking the country effect into effects of individual-level characteristics. However, Zou et al. (2009) argued that key cultural differences are carried by differences in individuals’ perceptions of their culture’s consensus beliefs, and proposed that individuals who perceive traditional views to be culturally consensual behave and think in culturally stereotypical ways. Therefore, examining values and preferences that members perceive to be widespread in their culture concerning workplace choice remains another topic for future research.

As a final future direction, it would be interesting to examine how preferences shift in response to changes in labor markets and their predominant organizational characteristics. In Japan in particular, major market reforms have recently been undertaken as various leaders try to rescue the country from its current economic stagnation. Consequently, many Japanese firms have attempted to adopt a performance-based system, and abandoned the traditional seniority-based system. Whether Japanese youth are adapting to these changes, or by contrast are becoming even more conservative and interdependent, is an empirical question (Ishii and Uchida, 2016). Therefore, in future research, it is important to investigate how institutional and cultural change influence people’s workplace choice not only across cultures, but also within.

As the economies of the twenty-first-century become increasingly globalized and culturally diverse, it is crucial to understand cultural differences in workplace choice. In this study, we provide initial evidence for an underlying mechanism (i.e., individual differences in desire for control) responsible for divergent preferences in both payment systems (reward-based vs. seniority-based) and adherence to family opinions (i.e., choosing a workplace consistent with one’s career aspirations vs. choosing a workplace consistent with the wishes of one’s family). Our results suggest that cultural differences in the psychology of choice, rather than being limited to inconsequential decisions, may be generalizable to decisions of vast importance such as the choice of where to spend one’s days – the choice of one’s workplace.

## Author Contributions

CE and KI designed research. CE, KI, YM, XM, and HH performed research. CE and KI analyzed data. CE, KI, and YM wrote the paper.

## Conflict of Interest Statement

The authors declare that the research was conducted in the absence of any commercial or financial relationships that could be construed as a potential conflict of interest.

## Footnotes

1 It can be assumed that one’s preference for the performance-based payment system and independence of parents’ opinions in one’s workplace choice are positively correlated. Indeed, we found a significant correlation, *r*(293) = 0.13, *p* = 0.03. The tendency was found in Germans, *r*(82) = 0.22, *p* = 0.04. However, the correlation did not reach a significant level in Japanese, *r*(79) = 0.05, *p* = 0.66 and in North Americans *r*(128) = 0.08, *p* = 0.37.

2 As in Study 1, one’s preference for the performance-based payment system was significantly positively correlated with independence of family opinions in one’s workplace choice, *r*(341) = 0.21, *p* < 0.001. The correlation was significantly positive in Japanese, *r*(104) = 0.26, *p* = 0.007, and in North Americans, *r*(121) = 0.19, *p* = 0.04, whereas it was not significant in Germans, *r*(112) = 0.11, *p* = 0.24.
