# Supplementary material for: To Accept One’s Fate or Be Its Master: Culture, Control, and Workplace Choice
Source: Front Psychol. 2016 Jun 21;7:936. doi: 10.3389/fpsyg.2016.00936 (PMC4914556; doi:10.3389/fpsyg.2016.00936)
Supplement: Supplementary file 1 [file Presentation_1.PDF]

Appendix A
Vignette and Questions concerning Payment System Preferences

Vignette: Imagine you applied for many jobs and first got many rejections but finally received two offers. Now you can choose. Both companies offer you a nice working environment and stable working conditions. You do not need to move since both companies are within one hour from where you are currently living. Both companies produce well-known products that interest you. They differ in their payment systems. One company offers you a seniority-based payment system. It pays a fixed salary that is enough to live on and you will receive a pay rise every 5 years. The other company offers you a performance-based payment system. You can get a pay rise if you fulfill your boss' expectations.

Choice question: Do you choose the job with the seniority-based payment system or the job with the performance-based payment system? Please select 1 if you definitely choose the company with seniority-based payment system or 9 if you definitely choose the company with performance-based payment system. If you are undecided, please select one of the remaining numbers indicating the likelihood that you would choose one or the other.

Reasons for the choice: In the following, you will find reasons for your above choice. Please select a number indicating your level of agreement to these reasons (1 = Strongly disagree, 9 = Strongly agree).

**1. The seniority-based payment system gives me a feeling of security.

*2. The performance-based payment system motivates me.

*3. Since I do not want to work all my life for the same company, I can get more money under the performance-based system.

**4. Under the seniority-based payment system, I know about the payment raise in advance and can plan my life accordingly.

**5. I don’t trust in my ability to improve my working performance. Therefore, the seniority-based system, which does not mirror my performance directly, is better.

*6. I want to get a reward for good work.

---

* Independent reasons

** Interdependent reasons

Appendix B

Vignette and Questions concerning Family Influence

Vignette: Imagine that you got two job offers from different companies and you need to decide quickly. Both companies are offering you a stable job and an average salary. You will have to work 5 days a week and overtime work will be paid. One company aligns with your family's expectations and desires for you, so choosing that one will make them very happy. The other company deals with topics you are interested in, but your family does not understand these topics nor your interest in them so it does not meet their expectations.

Choice question: Do you choose the company your family agrees with or the company your family disagrees with? Please select 1 if you definitely choose the company your family agrees with or 9 if you definitely choose the company your family disagrees with. If you are undecided, please select one of the remaining numbers indicating the likelihood that you would choose one company or the other.

Reasons for the choice: In the following, you will find reasons for your above choice. Please select a number indicating your level of agreement to these reasons (1 = Strongly disagree, 9 = Strongly agree).

**1. My family knows what is best for me.

*2. I could try to make my family understand why I want to do something different from what they want.

*3. It is important for my future happiness that I make my own decisions about my

career.

**4. It is important to respect my family’s wishes.

*5. I am responsible for my own future.

**6. My family is the most important part of my life and I do not want to be in conflict with them.

---

* Independent reasons

** Interdependent reasons
